# Impact of Electronic Cigarettes on the Upper Aerodigestive Tract: A Comprehensive Review for Otolaryngology Providers

**DOI:** 10.1002/oto2.25

**Published:** 2023-02-17

**Authors:** Joanne Soo, Meena Easwaran, Elizabeth Erickson‐DiRenzo

**Affiliations:** ^1^ Department of Otolaryngology–Head & Neck Surgery Stanford University School of Medicine Stanford California USA

**Keywords:** aerodigestive, e‐cigarettes (e‐cigs), electronic nicotine delivery systems, head and neck, otolaryngology, vaping

## Abstract

**Objective:**

The use and effects of electronic (e)‐cigarettes (e‐cigs) are particularly relevant for otolaryngology providers as tobacco plays a major role in benign and malignant diseases of the upper aerodigestive tract. This review aims to (1) summarize the recent policies regarding e‐cigs and important patterns of use and (2) serve as a comprehensive resource for clinical providers on the known biologic and clinical effects of e‐cigs on the upper aerodigestive tract.

**Data Sources:**

PubMed/MEDLINE.

**Review Methods:**

We conducted a narrative review on (1) general information on e‐cig use and informative findings in the lower respiratory system and a comprehensive review on (2) the effects of e‐cigs on cell and animal models and the clinical implications of these products on human health as is relevant to otolaryngology.

**Conclusions:**

Although e‐cigs are likely less harmful than conventional cigarettes, preliminary research on e‐cigs suggest several deleterious effects including in the upper aerodigestive tract. Due to this, there has been increased interest in restricting e‐cig usage, particularly among the adolescent population, and caution in recommending e‐cigs to current smokers.

**Implications for Practice:**

Chronic e‐cig use is likely to have clinical implications. It is critical for otolaryngology providers to be aware of the rapidly changing regulations and use patterns regarding e‐cigs and how e‐cigs influence human health, particularly with regards to the upper aerodigestive tract, to accurately council patients regarding potential risks and benefits of use.

Tobacco has been implicated in 70% to 80% of head and neck malignancies^1^, ^2^ as well as benign diseases of the head and neck.^3^ The morbidity of tobacco‐related head and neck disorders is immense; patients are often left with deficits in voice and articulation, dysphagia, and disfigurement. There are over 60 well‐established carcinogens in tobacco^4^, ^5^ and a number of toxins, including ones not seen in combustible cigarettes, have been also found in electronic (e)‐cigarettes (e‐cigs).^6^, ^7^

In response to the rapid rise in youth nicotine use as a result of e‐cigs,^8^ also known as electronic nicotine delivery systems, as well as growing evidence of the substantial harm of e‐cigs, numerous government agencies have initiated policies to reduce the incidence of nicotine addiction including the banning of many flavors in “cartridge‐based” products as well as imposing limitations on nicotine content.^9^, ^10^, ^11^, ^12^, ^13^ Nevertheless, the prevalence of e‐cig use remains high,^14^ in large part due to the exemption of disposable e‐cigs and flavor enhancers from these policies.^15^ The growing use of e‐cigs has prompted a commensurate increase in research on their effects. On PubMed alone, the number of published articles on e‐cigs has increased from 372 in 2014 to 1132 in 2021 and there are hundreds of review papers on various aspects of e‐cig use. Significant headway has been made in assessing the effect of e‐cig components on the respiratory and cardiovascular systems,^16^, ^17^, ^18^, ^19^, ^20^, ^21^, ^22^ and the number of studies exploring the effects of e‐cigs on other organ systems has also recently grown.^23^, ^24^, ^25^, ^26^, ^27^, ^28^, ^29^, ^30^, ^31^, ^32^, ^33^, ^34^, ^35^, ^36^, ^37^, ^38^, ^39^, ^40^, ^41^, ^42^, ^43^, ^44^, ^45^, ^46^

Despite the increase in research on the effect of e‐cigs on cellular biology and human health, reports on e‐cigs as they directly pertain to the upper aerodigestive tract remain limited, particularly for benign disorders. Furthermore, the current rate of relevant research on e‐cigs outpaces the ability of most practicing clinical providers to review. To provide a comprehensive review of e‐cigs as it is most relevant for providers who manage the upper aerodigestive tract, the review serves two main objectives. First, we provide a broad overview on the latest policies regarding e‐cigs, patterns of use in the general and adolescent populations, and highlight important insights on e‐cigs from the lower respiratory system. Second, we detail the current findings on the effects of e‐cigs on preclinical models and clinical outcomes in otolaryngology and identify areas and topics that require further research.

## Methods

To address our two distinct goals of (1) providing a broad up‐to‐date overview of e‐cigs and (2) comprehensively reviewing the direct impact of e‐cigs on the upper aerodigestive tract, we performed our review in two phases.

To address our first goal, we evaluated recent statements and resources regarding e‐cigs from the CDC, FDA, and the AAO‐HNS to determine which topics would be of greatest interest and relevance to the otolaryngology practitioner. Based on these, we decided to include in the review information regarding regulatory policies, use patterns, toxicities, and major known biological effects on human health. We then searched PubMed and Ovid Medline for articles published on e‐cigarettes in the last 10 years related to these topics. We additionally searched Google for rulings, policy statements, and news reports regarding e‐cigs.

**Figure 1:**
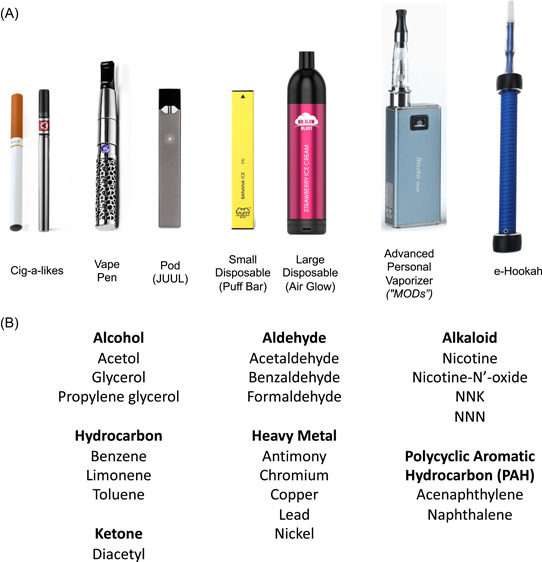
Common e‐cigarette (e‐cig) products and associated toxins. Representation of the (A) range of popular e‐cig devices and (B) known toxins found in e‐cigs.

Subsequently, to provide a comprehensive review of preclinical and clinical studies pertaining specifically to the upper aerodigestive tract and address our second goal, we performed a structured review of the preclinical and clinical studies by using a combination of the following search terms: e‐cigarette and/or otolaryngology and/or ENT and/or head and neck and/or oral and/or mouth and/or hypopharynx and/or oropharynx and/or nasal and/or nose and/or sinus and/or larynx and/or throat and/or voice and/or ear and/or otology. In addition, the reference lists of selected articles as well as review papers resulting from these searches were evaluated for additional studies not originally found. We included all primary literature that utilized cells, animal models, or clinical samples to evaluate the biological effects of e‐cigs in the upper aerodigestive tract. Studies were excluded if one of the following applied: not available in English, did not contain original data, or aims not relevant to biologic consequences of e‐cigs on the upper aerodigestive tract or ear (eg, articles assessing perceptions, policy, behavior, bronchial effects) including studies specific to dental health. All searches were performed in September 2021. Further details regarding searches conducted for goal 1 and goal 2 can be found in Supplemental Tables 1 and 2 and Figure 1, available online.

## Discussion

### Part One: Overview of E‐cigarettes

#### History and Marketing of E‐cigs

E‐cigs and other vaping products work by heating nicotine‐containing “e‐juice” or “e‐liquid” into an aerosol which is inhaled by the user. E‐juice is primarily made up of glycerin and propylene glycol into which nicotine, flavoring, and a range of other minor components are dissolved. These products not only go by several names but also come in a wide range of shapes and sizes (Figure 1A). Early “cig‐a‐likes,” introduced to the US market in 2006, simulated the size and shape of conventional cigarettes. Subsequently, cartridge/pod devices became more popular, particularly with the introduction of JUUL in 2015. Over the last few years, partially in response to government regulation on pod devices such as JUUL, disposables (eg, Puff Bar), and high‐powered advanced personal vaporizers or MODs have seen a dramatic increase in use.^47^, ^48^

E‐cigs are produced by both startup companies as well as major international tobacco companies and have been marketed as being different from conventional cigarettes, including with claims that e‐cigs are safe and benign.^49^ The perception among the general public is that e‐cigs use results in limited or no health consequences,^50^, ^51^, ^52^, ^53^ particularly flavored e‐cigs.^54^

#### Toxic Constituents in E‐cigs

Tobacco is known to have over 60 well‐described carcinogenic agents and hundreds more under study.^4^, ^5^ Although e‐juice has fewer known carcinogens as compared to combustible cigarettes, studies have proven the presence of toxic constituents in e‐cigs.^55^, ^56^ These constituents include but are not limited to carbonyl compounds,^57^ thiocyanate,^58^ heavy metals,^7^, ^59^, ^60^ silicate particles,^60^ diacetyl,^61^ and the known carcinogens formaldehyde^62^, ^63^ and *N*′‐nitrosonornicotine^56^, ^64^ (Figure 1B). Many of these components of e‐cigs come from the flavoring additives or devices themselves and thus vary widely between products.^65^, ^66^, ^67^, ^68^, ^69^, ^70^, ^71^ Several of these toxins have not been notably associated with traditional cigarette smoke. Bystanders can also be exposed to toxins in secondhand and thirdhand smoke from e‐cigs.^72^, ^73^, ^74^, ^75^

#### Regulation of E‐cigs

The largest concern regarding e‐cigs is the rise in new smokers, particularly adolescents, and relapsed former‐smokers.^8^, ^76^, ^77^, ^78^, ^79^ Once addicted to nicotine via e‐cigs, many adolescents explore other tobacco products and are more likely to use traditional combustible cigarettes.^80^, ^81^, ^82^ Due to the increased prevalence of e‐cig use, there has been progress in regulatory oversight for e‐cigs including the banning of many flavors in pods by the FDA^9^, ^10^ and capping of nicotine at 20 mg/mL in Europe.^11^ Most recently, the FDA has denied market authorization to the largest cartridge‐based producer, JUUL,^12^ though this has been stalled by a federal appeals court.^13^ Nevertheless, many methods including disposable and refillable e‐cigs remain largely unregulated.^15^ There is no production standard among manufacturers and thus the contents and potential toxicities can vary widely.^55^, ^56^, ^57^, ^58^, ^59^, ^60^, ^61^, ^62^, ^63^, ^64^, ^65^, ^66^, ^67^, ^68^, ^69^, ^70^, ^71^ Furthermore, users are increasingly creating their own device modifications, many of which pose an increased risk for elevated nicotine and carcinogen consumption and combustion.^83^, ^84^, ^85^, ^86^, ^87^, ^88^, ^89^, ^90^, ^91^, ^92^, ^93^, ^94^

#### Evaluating Patterns of Use

**Figure 2:**
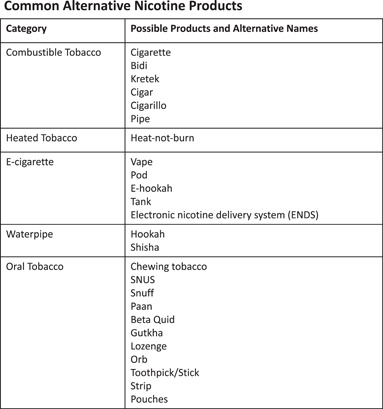
Range of alternative nicotine products. Patients who use alternative forms of nicotine, such as those listed, often deny a smoking history but will report use when asked about specific methods of consumption.

E‐cigs now make up the majority of nicotine use in teenagers and young adults^95^ and the largest portion of non‐cigarette or cigar use in older adults.^96^ In order to adequately counsel at‐risk patients about e‐cigs, medical providers rely on patients' self‐reported nicotine use. Studies have shown that the majority of users of alternative nicotine products including e‐cigs will answer “no” if asked about a smoking history but will affirm use of a particular alternative product when asked specifically.^97^, ^98^ While e‐cigs make up the largest share of alternative nicotine product use, there is a wide range of both traditional and modern alternative nicotine products including heated tobacco (HT) and oral nicotine products (ONP) (Figure 2).^99^, ^100^, ^101^ Use patterns also vary based on nationality and culture.^102^, ^103^ It is important, then, to ask about specific forms of tobacco consumption, both inhaled and smokeless, when assessing a patient's tobacco history. This is relevant for assessing both disease and surgical risk; an association with e‐cig exposure and poor wound healing and flap failure has been reported in both animal models and surgical patients.^97^, ^104^, ^105^, ^106^, ^107^, ^108^, ^109^, ^110^, ^111^ Unfortunately, due to the variable nicotine content of e‐cig products, it can be difficult to obtain a quantitative estimate of usage (ie, pack year equivalent).

#### E‐cigs for Current Smokers: Weighing Benefits Against Risk of Dual Usage

One popularly cited benefit of e‐cigs use is that they can reduce or eliminate the use of traditional cigarettes in current smokers. This potential benefit is the main reported driving factor for increased e‐cig use among cancer patients.^112^ There is indeed evidence that e‐cigs are not as harmful as conventional cigarettes.^113^, ^114^, ^115^, ^116^ However, the clinical impact of e‐cig use in current smokers is complicated by additional factors. Although e‐cig use has been shown to modestly reduce cigarette consumption,^117^, ^118^, ^119^, ^120^, ^121^, ^122^, ^123^, ^124^, ^125^, ^126^, ^127^ about 40% to 50% of smokers who do try e‐cigs become “dual‐users” who continue to smoke cigarettes but use e‐cigs in places smoking is prohibited, thus facilitating their addiction.^128^, ^129^, ^130^, ^131^, ^132^ Dual users often have a higher consumption of nicotine and toxic byproducts and lower rates of smoking cessation.^122^, ^133^, ^134^ Furthermore, a large portion of e‐cig users believe they are not addicted to nicotine despite regular use^52^, ^135^ which can limit motivation to quit. Therefore, while e‐cigs may be a useful tool for harm reduction in current smokers who are unwilling to otherwise reduce the use of combustible cigarettes or have failed other forms of smoking cessation, care should be taken to caution these patients about the risk of dual usage. This is reflected in the position statement by the AAO‐HNS regarding e‐cigs.^136^

#### Biological Impact of E‐cigarettes: Insights from the Lower Respiratory System

**Figure 3:**
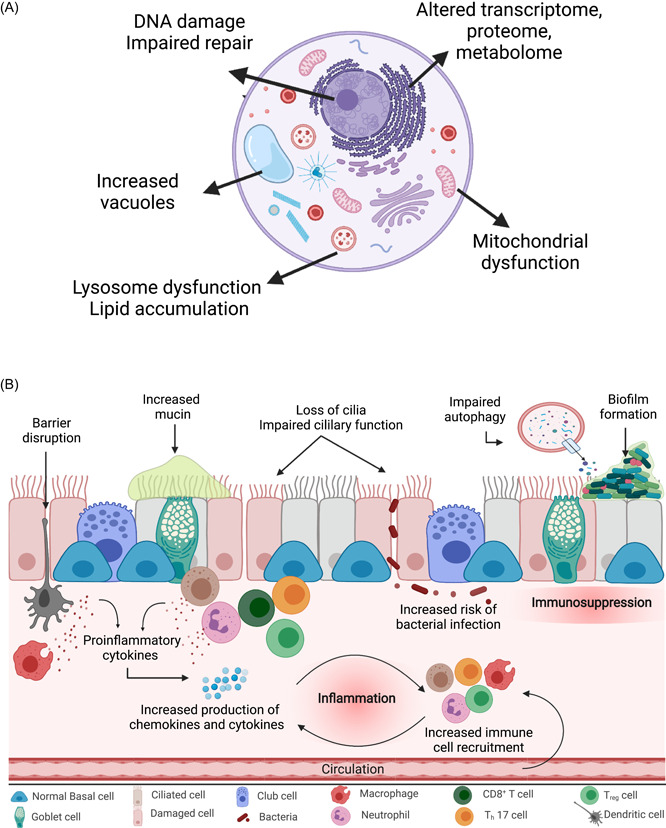
Cellular toxicity from e‐cigarettes (e‐cigs). (A) Example of pathways through which e‐cigs cause cytotoxicity. (B) Changes noted cell and animal models after e‐juice and e‐cig aerosol exposure.

E‐cigs have been studied extensively with regards to the lower respiratory system. Components of e‐cig aerosol have been shown to cause oxidative damage,^137^, ^138^, ^139^ DNA breaks,^140^ and inhibit DNA repair^6^, ^141^ in human‐derived lung cells which theoretically could lead to the development of oncogenic mutations (Figure 3A), though the effect size of mutagenic activity from e‐cigs is still unclear.^142^, ^143^ Several studies in lung‐derived cell lines demonstrate that e‐cig vapor results in gene expression changes similar to cigarette smoke and increases the risk of malignant transformation and metastasis.^6^, ^141^, ^144^, ^145^ E‐cigs have also been shown to cause mitochondrial dysfunction,^140^, ^146^ alter the cellular metabolome and proteome,^147^, ^148^, ^149^ and reduce cellular viability,^150^, ^151^ indicating that e‐cigs may not only increase the risk of cancer but also lead to the development of benign disorders. Finally, e‐juice and e‐cig aerosols have been demonstrated to potentially increase the risk of infection by promoting inflammation while reducing innate immune activity and the production of antimicrobial proteins.^152^, ^153^, ^154^

The effect e‐cigs have on cellular and tissue function has been confirmed in organoid and animal models (Figure 3B). Exposure to e‐cig aerosol and e‐juice has been shown to cause a wide variety of biological changes in the airway including increased production and decreased clearance of mucin,^155^, ^156^, ^157^ reduction in ciliary function,^157^, ^158^ increase in cytokines,^153^ and impaired immune cell function.^154^, ^159^ In respiratory models, e‐cigs have additionally been shown to increase alveolar‐capillary barrier permeability^160^ and increase airway hyperreactivity.^156^, ^157^ Finally, early data in mouse lung models demonstrates that prolonged e‐cig exposure can directly induce malignancy.^161^

Clinically, e‐cigs have been shown to directly impact lower airway epithelial cells in a similar manner as conventional cigarettes resulting in functional changes such as increased airway resistance and airway obstruction, symptomatic immune suppression, mucin production, irritation and inflammation, and lung disease exacerbation.^16^, ^21^, ^22^, ^162^, ^163^ While the risk of cancer in e‐cig users has not been fully studied due to the relatively short time e‐cigs have been on the market, preliminary experimental in vivo human data has demonstrated epigenetic changes in the airway after even brief e‐cig aerosol exposure including changes in the p53 pathway.^164^ In addition, over 2600 cases of e‐cig or vaping product use associated lung injury have been reported secondary to vaping in the United States alone,^18^, ^19^ largely due to the presence of vitamin E acetate in aerosolized cannabis oils.^165^

### Part Two: Biological and Clinical Endpoints in the Upper Aerodigestive Tract

#### Basic Research on E‐cigs in Otolaryngology

Several studies have evaluated the effect of e‐cigs in cell, organoid, and animal models of the upper aerodigestive tract (Table 1). The largest volume of primary literature on the effects of e‐cigs on the upper aerodigestive tract is in relation to oral and dental health.^26^, ^27^, ^28^, ^29^, ^166^, ^167^, ^168^, ^169^ As expected, e‐cigs do not appear to be as harmful as conventional cigarette smoke in cell and animal models.^170^, ^171^ Nevertheless, numerous studies have demonstrated biological disruption as a result of even brief e‐cig exposure.^29^ Liquid nicotine has been shown to promote the migration of dysplastic oral keratinocytes, suggesting a contribution of nicotine alone to oral carcinogenesis.^172^, ^173^ In the larynx, rats exposed to e‐cig aerosols showed early evidence of hyperplasia and metaplasia of the mucosa,^174^ although the results were not statistically significant in this limited study, and increases in IL‐4.^175^ In addition, both nicotine‐containing and nicotine‐free e‐juice have been found to cause biologic disruptions including oxidative stress, DNA breakage, metanuclear anomalies, liposomal dysfunction, and solvent and lipid accumulation, and cytotoxicity in human gingival fibroblasts,^67^, ^176^, ^177^, ^178^, ^179^, ^180^ vocal fold epithelial cells and fibroblasts,^181^, ^182^ head and neck squamous cell carcinoma cell lines,^67^, ^183^, ^184^, ^185^ noncancerous oral and oropharyngeal epithelial cells,^139^, ^178^, ^186^, ^187^, ^188^, ^189^, ^190^, ^191^, ^192^ middle ear epithelial cells,^193^, ^194^, ^195^ nasal epithelial cells,^196^, ^197^ and organotypic cultures/organoids.^178^, ^182^, ^197^ Interestingly, a recent study found that oral cancer cells exposed to e‐cig aerosols increased cell resistance to cisplatin through changes in drug transporters, suggesting a mechanism for e‐cig‐induced chemotherapy resistance.^198^ Therefore, the potential mutagenic and functional effects of e‐cigs and other noncombustible nicotine products on various cell types throughout the head and neck should not be underestimated.

**Table 1:** Primary Studies on the Effects of E‐Cigarettes on the Upper Aerodigestive Tract and Ear in Cell and Animal Models

References	Subject of interest	Study design	Exposure time (method)	Outcome(s)
Alanazi et al^177^	Primary human gingival fibroblasts	Ex vivo	1 hour a day for 1, 3, 5, and 7 days (extract in media)	Changes in morphology, decreased viability, increased apoptosis, delayed migration/wound closure
Carson et al^197^	Primary human nasal epithelial cell 3D model	Ex vivo	100 seconds (puffs over air‐liquid interface)	Reduction in ciliary beat frequency, development of fibrillar mesh‐like debris
Duggar et al^187^	Oral keratinocytes	In vitro	24 hours a day for 1, 2, and 3 days (extract in media)	Decreased growth, increase in cytotoxin expression
Ganapathy et al^139^	Human oral keratinocyte cell line (POE9n), human oral SCC cell line (UM‐SCC‐1)	In vitro	1 hour every other day for 2 weeks (extract in media)	Increased oxidative DNA damage
Go et al^185^	Human middle ear epithelial cell line (HMEEC)	In vitro	24 hours (extract in media)	Decreased viability, increased autophagy and apoptosis, increased cytokines, increased mucin production, dysregulation of water channels
Ha et al^175^	Murine model (C57BL/6)	In vivo	31 minutes and 40 seconds a day, 5 days a week, for 16 weeks (whole body chamber)	Differential inflammatory cytotoxin levels
Iskandar et al^170^	Human buccal epithelial (EpiOral) 3D model	In vitro	28 minutes (puffs over culture)	No significant histopathologic changes, significant differential gene expression (especially inflammatory pathways)
Ji et al^188^	Human oral epithelial cell line (NHOK)	In vitro	24 hours (extract in media)	Increased cytotoxicity, increased oxidative stress
Ji et al^189^	Human oral epithelial cell line (NHOK)	In vitro	4 hours (extract in media)	Differential gene expression (especially unfolded protein response)
Lungova et al^182^	Human induced pluripotent stem cell (hiPSC)—derived engineered vocal fold mucosae	In vitro	24 hours a day for 7 days (extract in media)	Changes in mucosal structure, increased mucin clots, dysregulated cytokine production, lipid deposition within cytoplasm and intercellular spaces causing epithelial injury and remodeling
Martinez et al^181^	Human vocal fold fibroblast cell line (hVFF)	In vitro	24 hours (extract in media)	Increased cytotoxicity, no significant differences in gene expression
Manyanga et al^198^	Human oral and pharyngeal epithelial cell lines (UM‐SCC‐1, WSU‐HN6, WSU‐HN30)	In vitro	24 hours a day for 2 and 4 days (extract in media)	Increased viability in setting of cisplatin exposure due to increased cisplatin resistance
Pushalkar et al^185^	Human hypopharyngeal SCC (FaDu) and leukoplakia (MSK‐Leuk‐1) cell lines	In vitro	40 minutes (puffed over culture)	Increased inflammatory cytokines in setting of infection, increased infection efficiency
Rouabhia et al^190^	Primary human gingival epithelial cells	Ex vivo	15 minutes a day for 1, 2, and 3 days (puffs over culture)	Change in morphology, increased apoptosis, increased DNA fragmentation
Rouabhia et al^196^	Primary human nasal epithelial cells and 3D model	Ex vivo	15 minutes twice a day for 1, 2, and 3 days (puffs over culture)	Changes in morphology, decreased viability, changes in mucosal structure, increased inflammatory cytokines
Sancilio et al^176^	Primary human gingival fibroblasts	Ex vivo	6 and 24 hours a day for 1, 2, and 3 days (extract in media)	Changes in morphology, decreased viability, increased apoptosis, increased ROS formation
Sancilio et al^179^	Primary human gingival fibroblasts	Ex vivo	3 and 24 hours a day for 1 and 2 days (extract in media)	Changes in morphology, increased cytotoxicity, changes in collagen release, increase in lysosomes, increased LC3 IIB/LC3 I expression
Salturk et al^174^	Murine model (Wistar albino, female)	In vivo	60 minutes a day for 4 weeks (whole body chamber)	Nonsignificant trend toward increased squamous metaplasia and hyperplasia, no difference in Ki67 expression
Song et al^193^	Human middle ear epithelial cell line (HMEEC‐1)	In vitro	24 hours (extract in media)	Decreased viability
Song et al^194^	Human middle ear epithelial cell line (HMEEC‐1)	In vitro	24 hours (extract in media)	Decreased viability, increased inflammatory cytokines, differential gene expression
Sun et al^191^	Human oral leukoplakia cell line (MSK‐Leuk1)	In vitro	16 hours (extract in media)	Increased metabolism of polycyclic aromatic hydrocarbons to genotoxic products
Sundar et al^178^	Human gingival epithelium cell line (HGEPp) and 3D model (EpiGingival)	In vitro	15 minutes (puffed over culture)	Increased protein carbonylation, increased inflammatory cytokines, increased DNA damage
Tellez et al^186^	Human oral epithelial (MOE1A, MOE1B) and leukoplakia (MSK‐Leuk1) cell lines	In vitro	20 minutes (puffed over culture)	Increased cytotoxicity, increased DNA damage
Tsai et al^184^	Human oral SCC cell lines (Ca9‐22, CAL‐27)	In vitro	24 hours (extract in media)	Altered cell invasion, increased RAGE expression, differential cytokine expression
Ureña et al^67^	Human oral SCC (SCC‐25) and gingival fibroblast cell lines (HGF‐1)	In vitro	3 minutes total—1 minute every 3 hours (puffed over culture)	Decreased viability, increased oxidative stress
Vermehren et al^180^	Human gingival fibroblast cell line (HFIB‐G)	In vitro	15 minutes (puffed over culture)	Decreased proliferation, increased metabolic activity, no significant difference in apoptosis or ROS formation
Welz et al^192^	Primary human pharyngeal organoid	Ex vivo	24 and 2.5 hours per day for 5 days (extract in media)	Increased cytotoxicity, increased DNA fragmentation
Yu et al^183^	Human oral SCC cell lines (HN30, UMSCC10B)	In vitro	24 hours a day for 1 week (extract in media)	Increased DNA strand breaks, increased cell arrest, increased apoptosis and necrosis

#### Translational Research on E‐cigs in Otolaryngology

A smaller number of studies have looked at the effects of e‐cigs in human users (Table 2). In a preliminary study looking at the oral transcriptome of e‐cig users and cigarette smokers as compared to nonsmokers, e‐cig users showed a disruption of multiple molecular pathways that have been implicated in carcinogenesis, many of which overlapped with smokers but some of which were unique.^199^ Consistent with previous research in cells, an increase in inflammatory markers and changes in the gene expression and metabolome can be detected from saliva and mucosa collected from e‐cig users compared to nonsmokers.^200^, ^201^ Another study found evidence of inflammation and immune suppression in the oral mucosa of e‐cig users^185^ which is consistent with separate studies with similar findings in the nasal mucosa.^202^, ^203^ Finally, analysis of the saliva of e‐cig users has revealed the presence of carcinogens,^58^, ^64^ metanuclear abnormalities in oral cells,^204^ changes in antimicrobial properties,^205^ and possibly the oral microbiome.^185^, ^206^ The generalizability and clinical applicability of these results will become more evident as additional studies are performed.

**Table 2:** Primary Studies on the Biologic Effects of E‐Cigarettes on the Upper Aerodigestive Tract and Ear in Human E‐Cig Users

References	Subject of interest	Study design	Number of e‐cig users	Outcome(s)
Alqahtani et al^200^	Saliva	Cross‐sectional	14	Elevated inflammatory cytokines, differential expression of metabolites
Bustamante et al^64^	Saliva	Cross‐sectional	16	Elevated levels of carcinogen *N′*‐nitrosonornicotine (NNN)
Cichońska et al^205^	Saliva	Cross‐sectional	40	Decreased lysozyme and increased lactoferrin, no difference in IgA
Flieger et al^58^	Saliva	Cross‐sectional	8	Elevated levels of thiocyanate
Franco et al^171^	Oral epithelial cells	Cross‐sectional	22	No significant increase in micronuclei
Hamad et al^201^	Buccal cells and blood	Self‐controlled	3	Differential gene expression
Martin et al^202^	Nasal epithelial cells	Cross‐sectional	12	Differential gene expression (especially suppression of immune‐related genes)
Pushalkar et al^185^	Saliva	Cross‐sectional	40	Altered oral microbiome, nonsignificant but considerable alterations in inflammatory cytokines
Rebuli et al^203^	Nasal epithelial cell	Clinical trial	15	Suppression of IgA in setting of viral inoculation, differential gene expression, differential cytokine levels
Schwarzmeier et al^204^	Oral epithelial cells	Cross‐sectional	20	Increased metanuclear abnormalities
Stewart et al^206^	Saliva and buccal swab	Cross‐sectional	10	No significant differences in oral microbiome
Tommasi et al^199^	Oral epithelial cells	Cross‐sectional	42	Differential gene expression

#### Clinical Research on E‐cigs in Otolaryngology

E‐cig use has also been associated with a wide range of self‐reported upper aerodigestive symptoms including throat irritation and discomfort, cough, tongue pain, nasal congestion, and sinus infections.^53^, ^207^, ^208^, ^209^, ^210^, ^211^, ^212^, ^213^, ^214^, ^215^, ^216^, ^217^ Although research on the effects of e‐cigs on human oral, oropharyngeal, and laryngeal health is still in its early stages, findings thus far are consistent with effects of e‐cigs in other organ systems and include immune suppression and inflammation (Table 3). Due to their effect on the immune system and oral microbiome, e‐cigs may additionally promote the risk of oral infection.^218^ Consistent with this, are multiple reports of inflammatory and infectious processes in the mouth and throat including uvulitis and epiglottitis which appear to be secondary to e‐cig use,^219^, ^220^ although interestingly there is a single case study reporting resolution of recurrent tonsillitis in a nonsmoker after e‐cig use.^221^ Finally, there are multiple reports of gingival and dental disease resulting from e‐cig use.^26^, ^27^, ^28^, ^29^, ^209^, ^222^, ^223^, ^224^

**Table 3:** Primary Studies on Clinical Symptoms and Findings in Patients Who Report E‐Cig Usage

References	Study design	Number of e‐cig users	Outcomes(s)
Andresen et al^233^	Case report	1	Fall with e‐cig in mouth resulting in pharyngeal and esophageal burns, diffuse supraglottic edema requiring tracheostomy and long‐term percutaneous feeding
Bardellini et al^225^	Case‐control	45	Similar total prevalence of oral mucosal lesions in e‐cig users compared to former smokers; increase in nicotine stomatitis, hairy tongue, and angular cheilitis compared to former smokers
Bartram et al^226^	Case report	1	Lichenoid eruption after switching from cigarettes to e‐cig with high propylene glycol content with near‐resolution after switching to e‐cig with low propylene glycol content
Bozzella et al^220^	Case report	1	Epiglottitis in e‐cig user without other risk factors or identifiable etiology; focal erosions/ulcerations and reactive/inflammatory changes on biopsy of arytenoid, soft palate, tongue
Brooks et al^90^	Case report	1	E‐cig explosion resulting in extensive intraoral and facial injuries
Brownson et al^85^	Case series	15	E‐cig explosion resulting in flame burns, chemical burns, and blast injuries
Cant et al^229^	Case report	1	Necrotic palatal ulcer in setting of e‐cig use
Cason et al^91^	Case report	1	E‐cig explosion resulted in extensive intraoral and facial injuries and inhalational injuries
Chen et al^215^	Observational	138,448	Wide range of self‐reported symptoms from e‐cig users, most commonly related to the respiratory system and mouth
Cho et al^209^	Cross‐sectional	216	Adolescent daily e‐cig users report increased rate of oral pain and dental concerns compared to never users
Demir et al^241^	Case report	1	Pediatric sudden sensorineural hearing loss after accidental ingestion of e‐liquid
Farinha et al^227^	Case report	1	Lingua villosa nigra after switching from cigarettes to e‐cig and resolution after switching back to cigarettes
Farsalinos et al^53^	Observational	19,414	Wide range of self‐reported symptoms from e‐cig users, most commonly sore/dry mouth and throat
Frossard et al^219^	Case report	1	Acute uvulitis after e‐cig use without other risk factors or known etiology requiring intubation
Gill et al^235^	Case report	1	Accidental ingestion of e‐liquid resulting in nicotine poisoning
Harrison et al^88^	Case report	1	E‐cig explosion resulting in extensive intraoral injuries
Huilgol et al^222^	Cross‐sectional	4957	Self‐reported daily e‐cig use associated with poor oral health compared to intermittent or no use
Hua et al^207^	Observational	481	Wide range of self‐reported symptoms from e‐cig users, most commonly related to the respiratory system and mouth/throat
Hua et al^208^	Observational	NA (41,216 posts)	Wide range of self‐reported symptoms from e‐cig users, most commonly related to the neurologic and respiratory symptoms and mouth/throat
Hughes et al^236^	Observational	256	Of 256 calls to a poison center regarding e‐cigs, majority involved children and the refill containers or fluid. Of pediatric patients who ingested e‐liquid, initial symptoms present in 32%
Jankowski et al^216^	Observational	61	Wide range of self‐reported symptoms from e‐cig users, most commonly cough and sore throat
King et al^210^	Observational	1624	Wide range of self‐reported symptoms from e‐cig users, most commonly cough and dry/irritated mouth/throat
Klawinski et al^231^	Case report	1	Stage IV SCC in 19‐year‐old with no other risk factors
Kumral et al^211^	Randomized control	42	SNOT‐22 scores worse in patients who used e‐cigs to quit smoking than those who quit without the use of e‐cigs
Kumetz et al^92^	Case series	2	E‐cig explosion resulting in extensive intraoral injury and burns
Li et al^213^	Cross‐sectional	641	Adult e‐cig users have increased wheezing and related respiratory symptoms compared to nonusers but less than current smokers
Luo et al^214^	Observational	NA (7927 posts)	Wide range of self‐reported symptoms from e‐cig users, most commonly respiratory and throat symptoms
Miler et al^221^	Case report	1	Resolution of chronic tonsillitis in a never‐smoker after vaping
Mokeem et al^218^	Cross‐sectional	30	Oral *Candida albicans* burden in e‐cig users is significantly higher than never‐smokers and similar to cigarette and waterpipe smokers
Moore et al^93^	Case report	1	E‐cig explosion resulting in intraoral injuries
Morse et al^84^	Case report	1	E‐cig explosion result in extensive oropharyngeal and palatal burns and associated right ear pain requiring intubation, Dobhoff tube, right SLN blocks, and extensive swallow and speech therapy
Nguyen et al^230^	Case series	2	Oral basaloid SCC in two patients with no other risk factors
Norii et al^94^	Case report	1	E‐cig explosion resulting in pharyngeal injury and C1 and C2 fracture
Reuther et al^104^	Quasi‐experimental	10	5 minutes of vaping in prior nonusers resulted in temporary increase in capillary perfusion of buccal mucosa
Richmond et al^237^	Observational	220	Of 220 cases of e‐cig inhalation and ingestion in children presenting to pediatricians, majority of inhalation in male adolescents while majority of ingestion was in male children. Both inhalation and ingestion most commonly resulted in nausea/vomiting, cough, throat irritation, or acute toxicity
Rogér et al^89^	Case report	1	E‐cig explosion resulting in extensive intraoral injuries and burns
Sample^239^	Cross‐sectional	7	E‐cig users had raised vocal shimmer compared to nonusers and use was associated with abnormal mucosal wave, free edge, phase closure, vocal fold varices, and vocal fold edema
Seo et al^234^	Case report	1	Death of a 15‐month‐old child from accidental ingestion of e‐liquid
Soule et al^212^	Observational	49	Wide range of self‐reported symptoms from e‐cig users, most commonly dry throat/mouth
Tsiouma et al^228^	Case report	1	Palatal ulceration in e‐cig user which resolved with treatment after switching to conventional cigarettes
Tuhanioğlu et al^238^	Cross‐sectional	21	No significant difference in VHI‐10 values or vocal quality between e‐cig users and nonusers
Vaught et al^86^	Case report	1	E‐cig explosion resulting in significant facial trauma
Walele et al^217^	Clinical trial	102	Prior smokers who switched from conventional cigarettes to e‐cig noted headache, nasopharyngitis, sore throat as the most common adverse events, most of which were transient

A few studies have demonstrated an increase in mucosal lesions in e‐cig users as compared to controls and former smokers.^225^, ^226^, ^227^ Two case studies have linked necrotic palatal ulcers with e‐cig use.^228^, ^229^ Importantly, multiple cases of oral carcinoma in the setting of prolonged e‐cig use in the absence of other risk factors have been reported.^230^, ^231^ Aside from effects of the toxic and carcinogenic contents of aerosolized e‐juice on the upper aerodigestive tract, e‐cig devices have also been implicated in multiple reports of burns and trauma of the upper aerodigestive tract and maxillofacial structures^83^, ^84^, ^85^, ^86^, ^87^, ^88^, ^89^, ^90^, ^91^, ^92^, ^93^, ^94^, ^232^, ^233^ and toxic ingestion.^234^, ^235^, ^236^, ^237^

Studies pertaining to the clinical effect of e‐cigs on the larynx and ear are particularly limited. One study evaluating acoustic voice changes in e‐cig users found no significant difference between e‐cig users and controls, however this study was limited by the small cohort size, short duration of e‐cig use (1‐3 years), and explicit exclusion of laryngeal pathologies for which cigarette and e‐cig exposure is known to be a significant risk factor such laryngeal inflammation and irritation.^238^ A second limited study also showed no change in acoustic measures between e‐cig users and nonsmokers but did find significant reduction in the vocal fold mucosal wave, irregularity of the free vocal fold edge, and abnormal phase closure as evaluated by videostroboscopy.^239^ While traditional cigarette smoke has been implicated in a number of otologic disorders,^240^ there is currently only one report regarding a case of sudden sensorineural hearing loss after ingestion of e‐juice^241^ with regards to the clinical effect of e‐cigs on the ear.

#### Future Directions

Although there has been an exponential rise in basic and translational work regarding e‐cigs in multiple organ systems, there are fewer studies pertaining specifically to the upper aerodigestive tract. Meta‐analyses on existing studies are needed but complicated by significant variations between and within studies in e‐cig brands, devices, and flavors used; exposure method; extract concentrations; and exposure durations; among other factors. Furthermore, the potential long‐term health consequences remain unknown; many consequences of cigarette smoking develop only after chronic use, and no current laboratory experiment can truly emulate decades of exposure. Even less is known about other alternative nicotine products including HTs and ONPs, which have been gaining market share and will likely play an increasing role in tobacco‐ and nicotine‐associated health concerns. While e‐cigs appear less carcinogenic than conventional cigarettes, the data thus far suggests a negative impact of e‐cigs across multiple organ systems including the upper aerodigestive tract. Given the outsized impact of smoking on diseases in otolaryngology, further work on e‐cigs and other alternative nicotine products is urgently needed.

Researchers in otolaryngology can benefit from building upon the research performed in models of other organ systems, particularly the lower respiratory system, to determine how e‐cig exposure interacts with the unique anatomy, physiology, and function of the various areas of the head and neck. In addition, the current reports on clinical diseases in e‐cig users pertaining to the upper aerodigestive tract are largely case studies and case series, thus necessitating further investigation to determine if e‐cigs are truly the causal agent. Finally, there are several avenues specific to otolaryngology that have been minimally explored, particularly the effects of e‐cigs on the sinonasal cavity, larynx, and ear.

#### Implications for Practice

Otolaryngology providers are among the first clinicians that nicotine users encounter with a smoking‐related disease process. Despite recent enhancement of policies to reduce youth nicotine use, youth use of and addiction to nicotine products has increased dramatically as compared to the decade prior, and medical practitioners should expect to see the health effect of this increase in the coming decades. In current smokers, the evidence for the utility of e‐cigs for overall risk reduction is heterogenous. While e‐cigs have been demonstrated to have fewer carcinogenic agents than their traditional counterparts—a fact often advertised by tobacco companies—there is evidence that these products remain a significant factor in the development of disease. Therefore, it is critical for every otolaryngology provider to stay abreast regarding the potential health consequences of e‐cigs, particularly with regards to the upper aerodigestive tract.

## Author Contributions

**Joanne Soo**, study design, literature search and analysis, manuscript preparation, revisions, approval for final version; **Meena Easwaran**, literature analysis, manuscript preparation, revisions, approval for final version; **Elizabeth Erickson‐DiRenzo**: study design, manuscript preparation, revisions, approval for final version.

## Disclosures

### Competing interests

None.

### Funding sources

None.

## Supporting information

Supplemental Figure 1.

Supporting information.

Supporting information.
