# Supplementary material for: Impact of Electronic Cigarettes on the Upper Aerodigestive Tract: A Comprehensive Review for Otolaryngology Providers
Source: OTO Open. 2023 Feb 17;7(1):e25. doi: 10.1002/oto2.25 (PMC10046796; doi:10.1002/oto2.25)

Goal 1: To provide a broad up-to-date overview of e-cigs

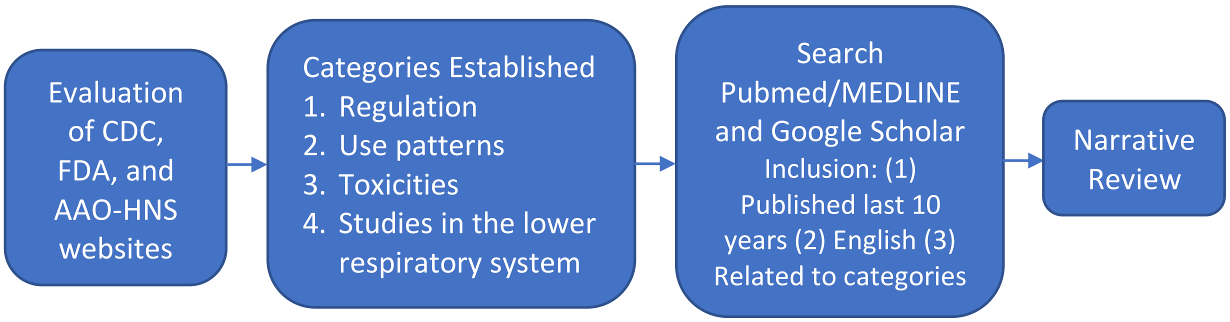

Goal 2: To provide a comprehensive review of the direct impact of e-cigs as is relevant to otolaryngology

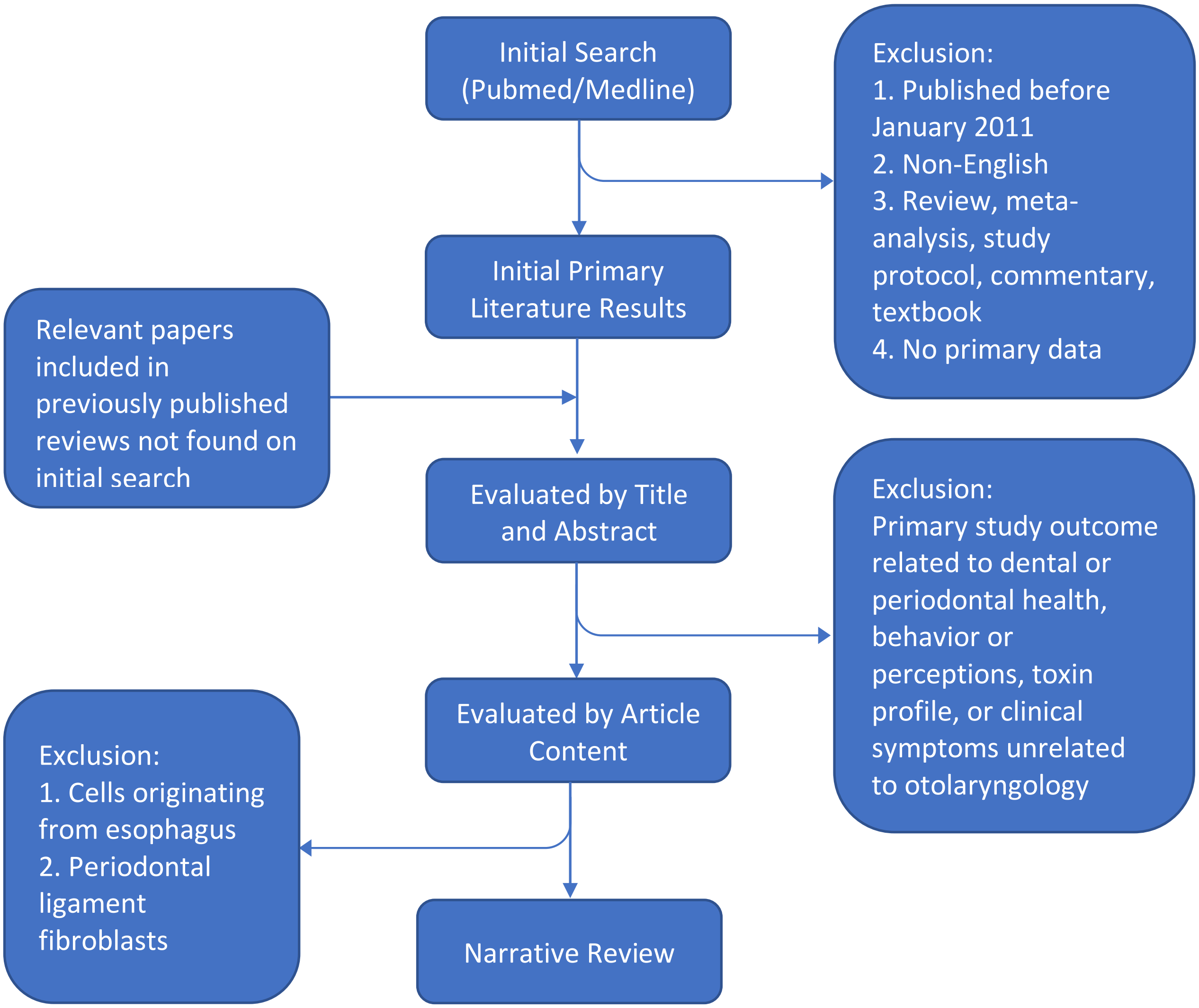

Supplement: Supplementary file 1 — Supplemental Figure 1. [file OTO2-7-e25-s002.pdf]
