# Supplementary material for: Impact of Electronic Cigarettes on the Upper Aerodigestive Tract: A Comprehensive Review for Otolaryngology Providers
Source: OTO Open. 2023 Feb 17;7(1):e25. doi: 10.1002/oto2.25 (PMC10046796; doi:10.1002/oto2.25)
Supplement: Supplementary file 2 — Supporting information. [file OTO2-7-e25-s003.docx]

**Supplemental Table 1.** Search terms used for preclinical and clinical studies related to otolaryngology.

| **Search term** | **Publications in PubMed/MEDLINE based on initial search** |
| --- | --- |
| “e-cigarette” AND “otolaryngology” | 30 |
| “e-cigarette” AND “ENT” | 4 |
| “e-cigarette” AND “head and neck” | 23 |
| “e-cigarette” AND “oral” | 126 |
| “e-cigarette” AND “mouth” | 51 |
| “e-cigarette” AND “hypopharynx” | 0 |
| “e-cigarette” AND “oropharynx” | 1 |
| “e-cigarette” AND “nasal” | 21 |
| “e-cigarette” AND “nose” | 11 |
| “e-cigarette” AND “sinus” | 4 |
| “e-cigarette” AND “larynx” | 1 |
| “e-cigarette” AND “throat” | 52 |
| “e-cigarette” AND “voice” | 13 |
| “e-cigarette” AND “ear” | 9 |
| “e-cigarette” AND “otology” | 1 |
