# Supplementary material for: Impact of Electronic Cigarettes on the Upper Aerodigestive Tract: A Comprehensive Review for Otolaryngology Providers
Source: OTO Open. 2023 Feb 17;7(1):e25. doi: 10.1002/oto2.25 (PMC10046796; doi:10.1002/oto2.25)
Supplement: Supplementary file 3 — Supporting information. [file OTO2-7-e25-s001.docx]

**Supplemental Table 2.** Inclusion and exclusion criteria for preclinical and clinical studies related to otolaryngology

| **Criteria** | **Inclusion** | **Exclusion** |
| --- | --- | --- |
| Publication Date | January 2011 – September 2021 | Before January 2011 or after September 2021 |
| Language | English | Non-English |
| Article Type | Primary literature | 1. Review 2. Meta-analysis 3. Commentary 4. Study Protocol 5. Textbook |
| Experimental Subject | 1. Human 2. Animal model 3. Cells and organoids originating from the upper aerodigestive tract or ear | 1. Periodontal ligament fibroblasts 2. Cells originating from the esophagus |
| Primary Outcome Category | 1. Cellular effects 2. Biologic effects in animal models 3. Biologic effects in humans 4. Clinical symptoms related to the upper aerodigestive tract and/or ear | 1. Dental outcomes 2. Periodontal outcomes 3. Behavior 4. Perceptions 5. Toxin profile 6. Clinical symptoms only related to other systems (e.g. evaluation only of wheezing, evaluation only of headaches) |
